# Student perceptions of a patient- centered medical training curriculum

**DOI:** 10.5116/ijme.536e.26b2

**Published:** 2014-05-25

**Authors:** Ashley Gallentine, Abraham A. Salinas-Miranda, Kathy Bradley-Klug, Emily Shaffer-Hudkins, Sara Hinojosa, Alicia Monroe

**Affiliations:** 1 College of Public Health, University of South Florida, USA; 2 College of Education, University of South Florida, USA; 3 Morsani College of Medicine, University of South Florida, USA

**Keywords:** Medical training curriculum, SELECT, patient-centered care, medical student focus groups

## Abstract

**Objectives:**

To evaluate a patient-centered medical training curriculum, the SELECT program, through perceptions of the inaugural student cohort.

**Methods:**

Data were collected from two focus groups conducted in the university setting, comprised of fifteen first-year medical students who participated in the SELECT program during its inaugural year. A questioning protocol was used to guide the focus group discussion, which was transcribed and hand-coded through thematic analyses.

**Results:**

Various themes related to patient-centered care were identified. Students noted changes in their attitudes towards interacting with patients in an empowering and educative manner as a result of communication and motivational interviewing exercises. Additionally, they recognized certain external, structural barriers as well as internal conflict between pragmatism and emotional intelligence that could potentially hinder patient-centered care. The impact of family dynamics and social support on quality of life and health outcomes was acknowledged. Students also emphasized the value of collaborating with multiple health professionals. Lastly, students provided suggestions for program improvement, namely additional simulations, more education regarding other healthcare professionals’ roles, more standardized experiences, and application of principles to acute and primary care.

**Conclusions:**

Upon completion of the first year of the SELECT program, students gained an appreciation for patient-centered care and various factors and skills that facilitate such care. Additionally, they experienced a dissonance between didactic concepts from the curriculum and observed medical practices. This study highlights the educational benefits of a patient-centered medical curriculum and provides suggestions for future improvement.

## Introduction

Medical student education is historically rooted in the diagnosis and management of illness and disease. Didactic content largely focuses on anatomy, pathology, pharmacology, and related coursework. Thus, while most graduates of medical school are prepared for medically diagnosing and treating illness and disease, little emphasis is placed on understanding how a patient’s lifestyle, beliefs, and behaviors impact his or her ability to care for and cope with a chronic illness.^1^,^2^Advances in medical care have decreased mortality and lengthened life expectancy for many individuals diagnosed with chronic health conditions. Such medical advances have thereby shifted the nature of many physicians’ work to the prevention and control of chronic conditions.^3^ The extensive impact of chronic illness on one’s life, daily functioning, and interpersonal relationships warrants the need for comprehensive care. Thus, health professionals must be familiar with and practice patient-centered care in order to attend to the broad needs of patients with chronic illnesses.^4^The Institute of Medicine defines patient-centered care as “providing care that is respectful of and responsive to individual patient preferences, needs, and values, and ensuring that patient values guide all clinical decisions.”^5^ Physicians operating under this model of care educate patients about their disease and potential treatments as well as develop an understanding of the entire patient, including cultural values, personal preferences, and their family and social situations. Physicians must use clear communication to build a patient-physician relationship in which common ground can be met regarding treatments and options.^6^ Moreover, given the known link between physical and psychological health, physicians and medical teams must acknowledge the potential value of assessing and monitoring quality of life for improving patient care and evaluating interventions.^7^,^8^ Patient-centered care has been correlated with positive perceptions from patients. Additionally, these perceptions were associated with positive health outcomes, such as better recovery from discomfort, better emotional health, and fewer diagnostic assessments and referrals.^9^

### The Scholarly Excellence, Leadership Experience, Collaborative Training (SELECT) medical student training program

The University of South Florida (USF) SELECT MD Program is a new model of medical education that seeks to instill and promote a patient-centered care approach among future physicians. The program emphasizes both personal and professional development through strategic mentoring and peer coaching, interprofessional and interdisciplinary team training, leadership skills training, and health systems and quality improvement training. These activities are designed to impart an added value to the core medical curriculum at the USF Morsani College of Medicine. Research indicates that emotional intelligence is highly associated with the characteristics and skills of effective leaders.^10^,^11^ The SELECT curriculum aims to foster the emotional intelligence of its students to train future physicians who are not only more engaged and compassionate, but also work effectively with teams and lead change in health care.In the first year of SELECT, students participate in didactic instruction focused on a variety of topics including health-related quality of life, interdisciplinary communication and collaboration, and values-based patient-centered care. Students learn specific skills such as active listening during clinical encounters, methods to assess for health-related quality of life, and motivational interviewing techniques for preparing patients to make lifestyle changes or adhere to treatment. Additionally, pairs of students are assigned to interdisciplinary clinical practices as part of a community-based clinical mentoring (CCM) program to observe all facets of patient-centered care. Each pair of students follows a patient from their clinical mentor’s practice to the patient’s home to get an in-depth picture of how health and/or illness impacts life outside the clinic visit. SELECT students are also given a comprehensive case study assignment designed to assess their knowledge and application of these constructs. The case study assignment was designed to assess students’ knowledge in the areas of interdisciplinary collaboration and communication, as well as assess their ability to use quality of life data to inform interventions, prioritize concerns, utilize available resources, and develop a plan to implement and monitor comprehensive interventions. Lastly, SELECT students participate in an action research project requiring them to meet with their clinical preceptors and collaboratively select an issue or topic of importance to investigate. The purpose of the action research project in the SELECT curriculum is twofold: to provide an opportunity for student doctors to learn about the application of research methods in applied settings and to complete an investigation focusing on outcomes that serve to inform and enhance their preceptor sites.To optimize the educational benefits of the program, review and evaluation of its various components is necessary. Feedback from students in the program is essential to an accurate and thorough program evaluation. The focus group methodology is particularly useful for gathering program evaluation data, particularly to capture rich experiential information from program participants.^12^,^13^ The purpose of the current study was to gather information from students in the inaugural SELECT cohort to understand the experiences and perceptions gained as a result of their involvement with the SELECT curriculum; and to understand their experiences and perceptions about different aspects of patient care and working with patients who are living with chronic conditions.

## Method

Two focus group interviews were conducted in July 2012 with student doctors from the inaugural class (2011-2012) of the SELECT program. These participants were contacted via email and asked to take part in a voluntary research study to evaluate their perceptions of the SELECT first year training curriculum and community-based mentoring. Students were told that the specific objectives of the study were to conduct focus groups designed to (1) evaluate the training curriculum from the perspective of the SELECT medical students, (2) evaluate the community-based mentoring experience from the perspective of the SELECT medical students, (3) identify potential challenges from the learners’ perspectives, and (4) problem-solve strategies to improve the training curriculum and/or the community-based experience for subsequent years.Participation in the focus groups was completely voluntary. No monetary or class grades incentives were offered. Students were told that their participation would not affect their status as a student. A written statement explaining the study, as well as the benefits and risks of their participation in the study, was attached to the email. Of the 19 students eligible to participate in the focus groups, 15 students volunteered, resulting in a 79% participation rate. No information was collected to ascertain why four of the eligible participants did not participate in the focus groups. Approval for this study was obtained from the university Institutional Review Board (IRB). Participants were provided with a written statement regarding the research and a waiver of informed consent documentation was approved by the IRB. This study asked participants to evaluate courses in the first year of a medical school program. This is a common practice at the end of courses in the medical school. Participants were able to respond in an anonymous manner and their answers were not linked to any one person, alleviating risk attached to expressing their opinions. A questioning protocol was used to guide focus group discussions (see Appendix A). Focus groups were conducted in English by two trained facilitators external to the College of Medicine. Each focus group lasted approximately one hour and the groups were held at a time of convenience in a designated classroom at the Morsani College of Medicine. Focus groups were audio-recorded and subsequently transcribed by a professional transcription company. Names were omitted in the transcriptions to assure confidentiality.Transcriptions and field notes were hand-coded by qualitative data analysts from USF through thematic analyses. A lead analyst initially created memos and thematic codes based on discussion topics identified and by reflecting on the study objectives. All coded textual data that related to each of the study objectives (domains) were grouped into emerging themes or categories. This process was verified by a second analyst for consistency and face validity.

## Results

A total of fifteen participants took part in both focus groups. Focus Group 1 had five male participants and three female participants, and Focus Group 2 had four male participants and three female participants. Both groups were diverse with respect to race/ethnicity. All participants were receiving financial aid to support their medical school expenses.The following is a summary of the combined findings from the two focus groups. The findings are organized by domain and include certain themes and subthemes that emerged as a result of the group discussions. The number of students that expressed the same perceptions or experiences were noted and are followed with representative quotes. Exact quotes were noted in text, put in double quotations marks, and italicized.

### Encouraging or affirming patient experiences

Students were asked what affirming or encouraging experiences over the past year changed their knowledge and attitudes toward patients living with chronic conditions. Two main themes emerged under this domain. The motivational interviewing learning module and communication exercises were conveyed by the students as being the source of their affirming or encouraging experiences.

#### Impact of motivational interviewing

The motivational interviewing exercises were encouraging experiences for the students and gave them the efficacy to apply these techniques in a clinical setting (ten students), resulting in affirming experiences, such as “The home visit was a reaffirming experience…getting to ask her questions [learn] of her quality of life” *(Female 1 FG2)*, “The patient interaction allowed you to readjust how you think about change, you want to find what motivates them… [help them] define health” *(Female 3 FG2)*. Several students described their realization that as upcoming practitioners, they had a responsibility to take on a more interactive role with patients (seven students). Student perceptions changed in how they approached the patient by helping the patient with the “decision-making process” and by learning what “their educational background is and where they are coming from” *(Male 4 FG1, Female1 FG 1)*. This role was further described in terms of helping the patient understand their condition and having the patient take control of their health, “the hands on the steering wheel versus the doctor [having] the hands on the steering wheel” *(Female 1 FG1)* and “work with the patient, not at the patient” *(Male 4 FG2).*

#### Impact of communication exercises

Several of the students agreed that communication can influence the type of relationship a patient has with their physician (nine students). For instance, effective communication in the choice and order of words was noted:

*“How you word things can affect how the patient perceives the relationship [with you] and can totally change their health outcome”* (Female 2 FG1).

The students did not realize the significance of communication with regard to developing patient relationships and enhancing positive health outcomes with their patient prior to SELECT: “My initial thought was face value, now I see how communication can impact the patient” *(Male 3 FG2).* Good communications with the patient allows the patient to feel like “they have control over their prognosis” *(Female 3 FG1).*Moreover, students explained how the communication exercises altered their perceptions of patients as non-compliant (three students): “My perception used to be that the patient was stubborn…now I understand that they are not educated on their disease or empowered to make a decision” *(Male 4 FG1)*. Students now see that communication can promote self-empowerment, education, and understanding on the side of the patient. This gives the patient the autonomy to make health decisions and feel empowered to comply “instead of giving up and giving in to their condition” *(Male 4 FG2).* Furthermore, students stated communication promotes long term relationships with their patients, which was perceived as important (five students).In summation, findings from this domain included changes in perceptions and attitudes towards patients in regard to how the student has a responsibility to educate, empower and develop interactive relationships with their patients. It appears the communication and motivational interviewing exercises from the SELECT curriculum had the most impact when changing these attitudes and gave students the knowledge on how to approach patients and develop an interactive relationship with them,

*“It’s nice to have a doctor there for each step and help them understand instead of just giving them a prescription to go fill”* (Female 3 FG2).

### Discouraging or unsettling patient experiences

Students were questioned about what had been the most discouraging or unsettling change in their perception and attitudes towards patients living with chronic conditions over the past year. The students perceived this question in two different ways, resulting in two different types of responses: internal reflection and external perception.

#### External reality of larger social and structural barriers

Many students stated the most discouraging or Students were asked to think about how their understanding of the family experience of persons living with chronic conditions developed over the past year. There was an array of responses to this topic that revolved around one main theme.Unsettling experience over the past year was learning about the external or environmental barriers that patients face. For example, two students stated their unsettling experiences pertained to patients that “wanted to receive treatment, but did not have the money to buy their prescriptions” *(Female 3 FG2).* A few others explained their discouraging experiences centered around watching the other “attendings” making assumptions about patients (three students). One student specifically stated,

*“I realized that not every attending received the training we received and really didn’t take into account the patients’ socioeconomic status or things that happened in their culture …they just write that the patient is non-compliant and don’t try to figure out the underlying reasons, it’s discouraging”* (Male 3 FG2).

#### Internal reflection of themselves as emerging practitioners

A few students (n=3) recognized they are essentially still pragmatic thinkers and tend to return to a “formula like thinking” *(Male 3 FG1)* and found this to be unsettling as they were progressing through the SELET curriculum. Internal reflection revealed they still revert to their “natural mathematical state of mind” *(Male 1 FG1)* whereas the SELECT curriculum is instilling a new way of thinking. The discouraging experiences occurred when they reverted back to that pragmatic thinking or went into a clinic full of patients with chronic diseases and had those initial assumptions about their patients.

*“It is difficult I want to jump right to what the pancreas is doing, and there is this [conflict] between the science and emotion”* (Male 1 FG1).

Even though these participants reported this line of thinking, they also reflected positively on the SELECT curriculum because they learned to question these assumptions as they arose. Students also mentioned when they get into the clinic they revert back to the “old guard” because they feel like people are telling them they do not have time to break the mold,

*“I feel like I fall in line with the old guard, you are trying hard to break the mold but everything around you is still like that and you have people telling you that you do not have time for this and that”* (Male 3 FG2).

In summary, the unsettling or discouraging experiences were split between some students’ internal reflection on their inner conflict between pragmatism and emotional intelligence; and other students’ realization of external forces that cause certain structural barriers for patients. However, the positive aspects of SELECT appear to have given most students a new way of viewing how patients should be approached and treated.

### Understanding the family experience

Students were asked to think about how their understanding of the family experience of persons living with chronic conditions developed over the past year. There was an array of responses to this topic that revolved around one main theme.

#### The importance of family dynamics in healthcare

A few of the students learned that being the “agent center” can be complicated and is different across settings:

*“It is difficult to balance between a mother and a patient, who do you ask the questions to?”* (Male 3 FG1)

Another student expressed the same difficulty with a patient who had an eating disorder:

“The interaction between the [family and the daughter] played a role and figuring out the dynamics was difficult…what are the family values about food?” *(Female 3 FG1)*

Other students (three students) expressed how healthcare can be time consuming for parents and how important the family dynamic is to positive health outcomes.

“They would bring in the whole family and everybody’s lives around that one person would have to change” *(Male 4 FG2).*

Another student explained that prior to SELECT she would have never actually focused on the family but just the disease itself. One student witnessed their mentor,

*“understood right away when she walked in the room that this was obviously a family dynamic problem…so she went directly to the problem, she did not even talk about food, she just talked about how they discussed these problems at home”* (Male 3 FG1).

Overall, SELECT played a role in changing the students’ perceptions of how remarkable and fragile family dynamics are in healthcare.

### Understanding the role of social supports

Students were asked to reflect upon how their clinical experiences impacted their perceptions of the role that social support and social networks play in managing chronic health conditions. Their responses demonstrated the connection between social supports and patient quality of life.

#### Role of social supports in quality of life

Each student identified that social support and social networks are significant to the “whole” treatment of the patient. Many students (eight students) recognized that a patient’s quality of life is directly related to and dependent on whether a patient is able to engage and maintain participation in activities that involve their support networks after diagnosis, or whether they have access to support groups and/or other community resources.

A few of the students reflected on their experiences in the NICU and all of them (three students) described how the “unit is stuck on survival mode” *(Female 1 FG1)* and,

*“no one has prepared the parents for what they are going to deal with, who is going to follow-up with them and make the appointments”* (Male 2 FG2).

These students all expressed concern about the lack of community resources and educational programming for parents and acknowledged how this will affect the quality of life for the entire family.Other students experienced patients that were disabled by their condition and were concerned with how they were going to engage in activities that allowed them to be active. The students identified that many of the patients were involved in hobbies that had a social aspect and their disease could affect their quality of life if they were unable to continue their involvement. One student observed:

*“getting the whole family onboard was…. key to getting the kid to exercise… so it is important to get the whole family excited about it”* (Female 3 FG2).

The overall experiences and perceptions relayed by the students encompass the understanding that social support and social networks are keys to the quality of life of a patient and patient treatment. Additionally, a discussion involving the low promotion of support groups and other community resources was noted.

### Interaction with medical professionals

Students were asked to think about how their interaction style with other medical professionals developed over the course of the curriculum. This topic also elicited two different types of responses; students either responded to how they interacted with their superiors or to how they experienced team collaboration in a clinical setting.

#### Student interactions with clinical supervisors

Certain students felt,

*“we are at the bottom of the totem pole…it is helpful to be aware of that. We are all tempted to jump in, but sometimes you have to ask permission before you can give an opinion or advice…take a step back from your ego.”* (Male 5 FG1)

This feeling was expressed by two other students. “It is important to know the appropriate time to speak up” *(Female 3 FG3).*

#### Interdisciplinary collaboration and professionalism

Students also responded to the way the SELECT program has prepared them for certain professional situations:

“One advantage of being in the SELECT program is that approaching these different issues with different professions is that I am able to approach it more systematically” *(Male 1 FG1).*

Students stated they felt SELECT gave them the tools to collaborate with other professionals and watch how medical teams communicate to provide optimal health care. Other students went further to say that,

*“SELECT allows us to recognize the differences between the good healthcare professionals and those that do not have the emotional intelligence”* (Male 3 FG2).

“I can definitely tell now who is there to provide for the patient and who is just there for the paycheck.” *(Female1 FG2)*

### Suggestions to improve the SELECT experiences

The students were asked to provide recommendations to the SELECT program for both the clinical and curriculum components. Students also provided general suggestions to improve the program for subsequent years. The following ideas were generated and categorized based on their responses.

#### Recommendations to enhance applied experiences

Suggestions for the clinical component of their training included the opportunity to follow a patient continually throughout the year, or have multiple visits. In addition, students suggested that the clinical site preceptors be reminded that the students are in their first year of training so “they do not expect third-year” student knowledge.

#### Recommendations to enhance the curriculum

Suggestions for the curriculum component of training included more interdisciplinary learning opportunities, more opportunities to role-play, and community support resources to assist patients seen at the clinical sites. In regard to interdisciplinary learning, students presented ideas such as,

“team building workshops with other health students’ *resources to assist patients seen at the clinical sites. In regard to interdisciplinary learning, students presented ideas such as “team building workshops with other health students and other health systems”; “have mock teamwork with some of the nursing students and pharmacy students…we may be working with them one day…this would help network”.*

Requests were also made for more education about the function and responsibilities of other health professions, such as social workers and nurses. The students suggested more opportunities to role play by doing simulations for motivational interviewing with patients or having crucial conversations. They also suggested holding a “gown and prep clinic”, and “presenting” a patient clinic. Finally, students emphasized the utility of more knowledge of community resources for when they are interacting with patients. A suggestion was made to provide students with a list of community resources or educational programming for parents, as well as a list of locations for social support groups for patients. In addition, students requested instruction on nutritional guidelines and other talking points on diet and exercise for patients.General suggestions for improvement included more organization and a clearer idea of what is expected from the students as far as projects and other assignments. Students also expressed a desire for the curriculum to include acute care and primary care in addition to chronic care.Overall, the main recommendations from students centered on having additional simulations incorporated into the curriculum, and learning more specifics of the roles of other healthcare team members. The most agreed upon and discussed recommendation revolved around standardizing this experience across all the various domains of clinical learning, with support from other attendings and members of the healthcare team. The students highlighted the importance of taking these core concepts and applying them in other scenarios and different situations, such as acute care and primary care.

## Discussion

The purpose of this study was to gather student feedback on the inaugural year of the SELECT training program as part of the ongoing program evaluation of this curriculum. Focus groups using guiding questions were held with first-year medical students to provide an open forum for discussion. Themes generated from these focus groups provided insight into what the students view as program strengths and challenges, and what they recommend for future training opportunities.

### Program strengths

Students found the opportunities to interact with professionals from multiple disciplines at their preceptor sites to be a strength of the program. Specifically, students commented that they appreciated being able to observe the interactions between team members and they developed tools to facilitate collaboration. In many cases, the students requested additional opportunities for interdisciplinary interactions. Interestingly, they reported that observing professionals working together allowed them to better understand the concept of medical professionalism. They were able to identify situations where professionalism was evident and other instances where this type of behavior was lacking. Through these observations they were able to discern the value of professionalism and its impact on interdisciplinary collaboration and patient-centered care. The learning modules in motivational interviewing and communication were reported as the most helpful during this first year of training. Students stated that they were able to apply these skills directly in their clinical sites. It is of importance to note that both of these skill areas are directly tied to the core concepts of patient-centered care. By building trusting relationships with patients through clear, respectful communication and empowering patients to better understand their health condition, treatment plans incorporating patient values can be developed.^2^,^6^Students also stated that through the SELECT curriculum they learned to see a patient as a person and not just a disease. They developed an understanding of the importance of family dynamics especially as they relate to treatment outcomes, as well as the role of the social environment in providing support for patients. Students learned that a patient’s quality of life is not necessarily defined by a disease, but by a number of factors that may not be clearly evident to the physician during a brief physical examination. Once again, this understanding is critical to the concept of patient-centered care.^14^In addition to the most beneficial aspects of the program, students also provided feedback on how the curriculum was delivered. Specifically, consideration of adult learning styles is important as the students preferred opportunities to directly apply skills and knowledge from more didactic learning modules to actual patient interactions. Thus, the identification and recruitment of preceptor sites that offer experiences for these students to directly apply the skills they are learning is critical in the planning process. Chosen preceptor sites should utilize an interdisciplinary team approach to patient care and offer direct patient interaction.

### Program challenges

In addition to the strengths of the SELECT training program, students also reflected upon challenges they faced during this training. One of the challenges identified was the inconsistency between what they learned during their training at the medical school and what they experienced in the field. For example, some students reported that the physicians they shadowed engaged in practices that were contrary to what they were learning about professionalism and patient-centered care. These types of situations resulted in feelings of cognitive dissonance. Specifically, students were learning particular skills and behaviors that were expected of them in practice and yet observing behaviors by professionals in the field that were somewhat opposed to those behaviors. Although students shared their discomfort and frustration with these types of experiences, their ability to reflect on the disconnection between training and practice is viewed positively. In other words, the fact that they were able to observe these differences and experienced dissonance demonstrates that they have learned the importance of patient-centered care. It is our hope that this dissonance motivates the students to create change in the medical system, and that the leadership training portion of the SELECT program provides them with the skills to do so.Another challenge reported by students was based on the overall communication and organization of information provided to them about the training curriculum and student requirements. It is acknowledged that because this was the inaugural year of the training program, communication about activities and expected student products were not as timely as would have been preferred by students. This feedback is extremely helpful as the curriculum team prepares the curriculum and syllabi for subsequent training years and future cohorts.

### Training recommendations for the future

Feedback from these first-year medical students was very helpful in planning for the future of this training curriculum. Students almost unanimously requested additional opportunities for practicing the skills learned through role-playing and simulation exercises. The SELECT curriculum team has already taken steps to infuse these types of activities throughout all of the training modules. Additionally, more opportunities for interdisciplinary interactions have been added, including simulation and applied activities with students from other professional areas such as nursing, public health, physical therapy, and pharmacy. Mentoring opportunities have also been developed so that advanced doctoral students in public health and pediatric school psychology are available to provide support to first-year medical students as they complete their action research project competency. As mentioned previously, much of the student feedback pertains to the importance of finding preceptors and sites that model the values of the patient-centered care model and will allow students opportunities for applied practice and supervision.In general, the SELECT program provided first-year medical students with the skills and knowledge to learn and observe components of the patient-centered care model in practice. These findings indicate the program elicited social and emotional thinking as well as raised other questions surrounding the healthcare system and factors regarding patients as a whole, including the impact of one’s health on quality of life and relationships.

### Limitations

Results of the current study should be considered in the context of certain methodological limitations. During the analysis there appeared to be a difference in the way the questions were perceived by the students between the two focus groups resulting in a mix in the responses. There also appeared to be more responses from certain students than from other students and therefore saturation was not met under certain domains in this report. It is also possible that some SELECT students’ perspectives were not captured because only two focus groups were conducted.

## Conclusions

This study provides valuable insight into the educational benefits of the SELECT program and perceptions the students gained regarding patient-centered care and quality of life with chronic health conditions. The program enhancements resulting from the study findings demonstrate that student perspectives provide a key source of information for curriculum improvement. Further research could focus on assessing longitudinal change in perceptions during the years of medical school education and residency, as well as on patients’ perspectives of interactions and care delivered by healthcare professionals trained under the SELECT curriculum.

## 

### Conflict of Interest

The authors declare that they have no conflict of interest.

## Appendix A

### Focus Group Agenda

Introduction: Thank you for participating in this focus group. We are here to discuss your experiences and opinions about different aspects of patient care and the overall experiences of those living with chronic health conditions. The goal of today’s group is to engage your voices in the refinement and further development of curriculum and experiences for SELECT students. I have several questions for you regarding your ideas and experiences throughout your first year of medical school.

I will facilitate in a way that helps us cover all the questions the time we have together. I invite you to be as honest as possible. No identifying information will be connected with any content shared. I will occasionally go around the group and check-in with each person to make sure all voices are being heard. Please be mindful of your own talk time as well.

As you answer the following questions, please reflect on this past academic year of your medical school training, particularly in regard to the content you learned related to quality of life, team communication and collaboration, and patient-centered care. Consider the ways in which these concepts informed your thinking and how you applied these concepts during your community clinical mentoring (CCM) experience. This may include readings, class discussions, clinical experiences, or course assignments that you completed related to the concepts above.

First, reflect on your knowledge and attitudes about persons living with chronic health conditions before beginning this year of medical school. This may have been influenced by your personal experiences. Now, compare this to your current knowledge and attitudes.

1. Describe how your impression of the impact of a chronic health condition on persons with chronic illness has developed this year.

Specifically,

- What has been the most affirming or encouraging change?
- What has been the most unsettling or discouraging change?
  1. Describe how your understanding of the family experience for persons with a chronic health condition has developed this year.
  2. Describe how the CCM experience has impacted your understanding of the role that social supports and social networks play in managing a chronic health condition.
  3. Describe how your interactional style may have developed in the way that you approach and communicate with patients over this year. (e.g., how you talk with patients, how you listen)
  4. Describe how your interactional style may have developed in the way that you approach and communicate with other medical professionals over this year.
  5. If you were developing a curriculum or clinical experience for first-year SELECT students, what other kinds of skills or issues would you include? How would you design these experiences so that they would be most beneficial to students?
  6. Is there anything else you think we should know about your experiences with the first year of the SELECT program?
